# The Correlates of Credit Loss: How Demographics, Pre-Transfer Academics, and Institutions Relate to the Loss of Credits for Vertical Transfer Students

**DOI:** 10.1007/s11162-019-09548-w

**Published:** 2019-02-21

**Authors:** Matt S. Giani

**Affiliations:** The University of Texas at Austin, Austin, TX USA

**Keywords:** Transfer, Articulation, Credit transferability, Credit loss, Community colleges

## Abstract

Despite the long-standing centrality and growing prevalence of transfer in the American postsecondary system, students, college professionals, and policymakers decry the lack of credit transferability between colleges. However, limited research has examined the factors most related to the magnitude of credit loss students experience. This study investigated how students’ pre-transfer academic characteristics, demographic characteristics, and the institutions they transferred to and from influenced the magnitude of credit loss they experienced. Data is drawn from statewide cohorts of vertical transfer students in two states: Hawaii and North Carolina. Although a number of demographic and pre-transfer academic factors were found to relate to credit loss, the predictors of credit loss varied appreciably across states. Given the significant variability in how states and postsecondary systems manage transfer and articulation, the findings point to the need for additional state-level research exploring the determinants of credit loss for transfer students.

## The Correlates of Credit Loss for Vertical Transfer Students

Since their inception over a century ago, community colleges have served as a gateway to upward mobility for millions of students by allowing them to begin their postsecondary experience at a 2-year institution before transferring to a 4-year college and completing a bachelor’s degree. Indeed, prior to the widespread adoption of the term community college, most of these sub-baccalaureate institutions were called junior colleges and only offered students academic courses corresponding to the first two years of a bachelor’s degree so that students could “transfer these course credits to a senior college or university, at which point they would begin their specialized or professional studies” (Diener 1986, pp. 7–8). And although the mission and offerings of community colleges have expanded significantly over time to include far more technical and vocational pathways, the vast majority of students who begin at a community college intend to transfer to a bachelor’s degree granting institution (Laanan 2003). However, few community college students successfully transfer to a 4-year college (Jenkins and Fink 2016), and only half of students who do transfer eventually earn a bachelor’s degree (Monaghan and Attewell 2015; Shapiro et al. 2015).

A host of policies and practices have been developed to ensure ease of transfer, defined as “the mechanics of course, credit, and curriculum exchange” (Kintzer and Wattenbarger 1985). These include transfer and articulation agreements, transfer-oriented associate’s degrees, transferrable common cores, and common course numbering, among others (Millard 2014). Despite these strategies, of concern to policymakers, college practitioners, and students is the issue of credit loss, in which the receiving institution does not accept credits previously earned by the student. Indeed, research has shown that a significant proportion of students lose credits during the transfer process (US Government Accountability Office 2017; Monaghan and Attewell 2015; Simone 2014).

Although the issue of credit loss is known, less understood are the correlates of the magnitude of credit loss students experience. Specifically, limited research has neither investigated how factors such as students’ demographic characteristics and their pre-transfer academic experiences impact the magnitude of credit loss they experience, nor how the severity of credit loss varies across institutions. This study attempts to address this gap in the literature by exploring how credit loss varies by student characteristics and institutions across two states: Hawaii and North Carolina. These states both participated in the *Credit When It’s Due* (CWID) initiative, designed to award associate’s degrees to students who transfer from 2-year to 4-year institutions without the degree if they complete the degree requirements at the 4-year institution, and provided the CWID research team with extensive data on cohorts of transfer students that allowed for an investigation of credit loss.

## Prevalence of Credit Loss among Transfer Students

Research from the Education Commission of the States (ECS) shows that the majority of states have implemented policies designed to facilitate the transfer of students and credits between postsecondary institutions (Millard 2014). As of 2014, 36 states had policies ensuring that students who earn an associate’s degree will be able to transfer all of the credits related to their degree and enter with junior standing when they transfer to an in-state public 4-year institution, and 35 states had implemented a transferrable common core of lower-division courses that must be accepted by all public institutions in the state. Although less common, a number of states also use practices such as common course numbering and credit by assessment to facilitate the transfer of credits and recognition of prior learning.

Despite the prevalence of these policies, there is concern that the transfer process may not be as seamless as policymakers and higher education practitioners intend. There are three principal issues in this regard. The first is the increasing prevalence of non-traditional pathways through higher education and student “swirl” (Borden 2004; McCormick 2003; Townsend 2001). Recent cohorts are not only more likely to transfer than their peers in prior cohorts, they are more likely to complete multiple transfers, reverse transfers (4-year to 2-year), and lateral transfers (e.g., 2-year to 2-year) (Adelman 2006). Indeed, the term “traditional student” may be a misnomer given that the student who graduates from high school, enrolls directly in a postsecondary institution, attends only that institution, and earns a credential within the recommended timeframe is the exception rather than the rule. Data from the National Student Clearinghouse (NSC) shows that roughly one-third of students who begin college at a public 2-year institution complete at least one transfer within five years of their initial enrollment, and students who begin at a public 4-year institution actually have higher rates of transfer compared to students who begin at public 2-year colleges (Hossler et al. 2012). Additionally, students who begin at community colleges are almost as likely to transfer to another 2-year institution compared to a public 4-year (36.5% vs. 42.2% of transfers), and students beginning at public 4-years are more likely to transfer to a public 2-year than another public 4-year (38.1% vs. 35.7%). Thus, in addition to transfer becoming more prevalent, the transfer pathways students are pursuing have become more complex.

The second issue is the inability to transfer credits to a receiving institution. Three studies using data from the National Center for Education Statistics’ (NCES) Beginning Postsecondary Students (BPS) longitudinal survey have recently attempted to quantify the magnitude of credit loss experienced by transfer students. Simone (2014) estimated that less than one-third (32.4%) of students who transferred to or co-enrolled in another institution within six years of their initial enrollment were able to transfer all of their credits. These students earned an average of 24.2 credits at their origin institution before transfer. In contrast, 39.4% of the sample lost all of their credits upon transferring, and the remaining 28.2% of students lost at least some of their credits. Out of this subgroup who experienced partial credit loss, only 33.4 of the 46.3 credits their earned on average were able to transfer for an average loss of 12.9 credits (27.9% of credits earned), or roughly a full semester’s worth of credits.

The United States Government Accountability Office (US GAO) (2017) also used BPS data to analyze the rates of transfer and credit loss for students who transferred at least once between 2003–2004 and 2008–2009. Roughly 35% of beginning college students completed at least one transfer during this period, losing an average of 13 credits (43%), equivalent to roughly one semester of coursework. The US GAO also found that the magnitude of credit loss varied significantly based on the type of origin and destination institutions. Students who transferred from public 2-year to 4-year colleges, the traditional vertical transfer route, lost only 22% of their credits. In contrast, students transferring between for-profit and non-profit schools, whether 2-year or 4-year and whether the for-profit was the sending or receiving school, lost more than 90% of their credits on average. Interestingly, transfers between different public 2-year colleges lost an average of 69% of their credits, suggesting that credit transferability is not simply influenced by the public/private nature of colleges. The significantly lower rates of credit loss between public 2-year and public 4-year colleges likely reflects the impact of transfer and articulation agreements that exist between many public 2-year and 4-year colleges in the same postsecondary system.

It should be noted that both Simone’s analysis and the US GAO report included all beginning postsecondary students, regardless of initial institution of enrollment or the institution to which students transferred. Monaghan and Attewell (2015) used the same BPS dataset but restricted their sample to students who began at community colleges and transferred to a 4-year college. Although their analyses also showed a large prevalence of credit loss, the scope and severity of credit loss was not as extreme as Simone’s analysis. Monaghan and Attewell found only 14% of the sample lost 90% or more of their credits, 58% were able to transfer 90% or more, and the remaining 28% lost between 10% and 89% of their credits. Critically, these authors estimated that students who are able to transfer all of their credits have roughly 2.5 times the odds of earning a bachelor’s degree, even when controlling for pre-transfer GPA and the number of credits earned prior to transfer.

The third issue occurs when students are able to transfer credits to a receiving institution but are unable to apply transferred credits to their chosen degree plan. Although credit loss is occasionally described in the literature as the inability to transfer credits, the inability to apply credits may be an even larger issue. As Kisker et al. (2011) stated:

Policymakers and educators…must move beyond consideration of course transferability and focus instead on how credits will apply to specific academic and degree requirements at receiving institutions. (p. 3)

The challenge for researchers is that there are limited datasets that would allow for an empirical examination of “program credit loss” (see Hodara et al. 2017), as neither federal longitudinal studies nor state higher education agencies regularly collect college transcript data that specifies whether specific courses were applied to program requirements for degrees students completed.

One method by which researchers have attempted to approximate the magnitude of program credit loss is by comparing the credit accumulation patterns of transfer students vs. native 4-year students. Although to the author’s knowledge no national studies have taken this approach, a number of state-level analyses have estimated that transfer students generally accumulate more credits on their way to the baccalaureate compared to native students. Some studies find relatively modest disparities. The difference in total credits between transfer and native baccalaureate completers was estimated to be four credits in Washington (West 2015), eight credits in Texas (Cullinane 2014), and nine credits in Kentucky (Kentucky Council on Postsecondary Education 2008). However, a recent study using two anonymous states found that bachelor’s completers who started at a 2-year college attempted 153.8 credits on average compared to 129.1 credits for native students, a difference of approximately one year (24.7 credits) of courses (Belfield et al. 2017). Although not definitive, these findings are congruent with the hypothesis that students who transfer may not have all of their credits applied to their degree program.

Overall, the estimated prevalence and magnitude of credit loss varies widely according to the specific datasets used (e.g. national longitudinal survey vs. state data), the sample under consideration (e.g. all transfers vs. public 2-year to public 4-year transfers), and the manner in which credit loss is measured (e.g. transfer credits accepted vs. total credits earned at time of degree receipt). Nevertheless, the extant literature offers some support for the assertion that students lose from a modest to a non-trivial number of credits upon transfer, and transfer students often earn a higher number of total credits at the point of baccalaureate attainment compared to 4-year native students. The following section digs deeper into some of the potential mechanisms contributing to credit loss.

## Conceptual Framework: State, Institutional, and Student Level Correlates of Credit Loss

While a growing number of studies have sought to estimate the magnitude of credit loss students experience, there remains a dearth of empirical research examining factors associated with credit loss, and no conceptual framework has been proposed to identify the most salient predictors of credit transferability. This study therefore draws from other frameworks and literature on transfer and articulation in order to generate hypotheses about correlates of credit loss. The following sections discuss factors at the state, institutional, and student level that research suggests may be associated with credit loss.

## State Policy

Perhaps the most obvious potential source of credit loss is the absence or underdevelopment of transfer and articulation agreements, particularly state-wide systems governing transfer. As Roksa and Keith (2008) argued, “Statewide articulation policies, as specified in the state legislation, are not designed to facilitate transfer per se. Instead, articulation policies are designed to preserve credits as students move from two-year to four-year institutions” (p. 239). The governance structure of a state’s higher education system, such as whether the state has a governing or coordinating board for higher education and the strength of college and university systems (Nicholson-Crotty and Meier 2003), influences whether transfer policy will be primarily established by the state legislature, the higher education agency, college and university systems, or individual institutions. This has important implications for the potential robustness of transfer and articulation policy, such as the institutions covered by the policy (e.g. public vs. private, 2-year vs. 4-year), the components of the policy, and mechanisms for enforcing institutional compliance (Ignash and Townsend 2000, 2001). Policies such as common course numbering, common core courses, and transfer-oriented associate’s degrees may be part of a policy framework designed to prevent credit loss, but these frameworks may be voluntary or may give the state little recourse to enforce compliance even when they are mandatory.

Given this complexity, whether state transfer policies serve as a support the movement of students and credits between institutions is a continued source of debate. It is logical to presume that states with more robust and detailed transfer and articulation policies would have lower rates of credit loss and thus improved persistence and attainment outcomes among transfer students, controlling for relevant factors that could affect students’ postsecondary outcomes. However, research has found that states with transfer and articulation policies have roughly equivalent transfer student outcomes compared to states with no such policies. Roksa and Keith (2008) found that transfer students had slightly higher bachelor’s degree attainment rates in states with statewide transfer and articulation policy compared to their peers in other states (61% vs. 57%), but actually had longer average time-to-degree (5.50 vs. 5.42) and a greater number of credits earned (147 vs. 140). None of these differences were statistically significant in regression models controlling for students’ demographic and pre-transfer academic characteristics. These findings are echoed by related research showing a limited relationship between the presence of statewide transfer policy and actual transfer rates (Anderson et al. 2006; Kienzl et al. 2012). However, to the author’s knowledge, no study has conducted a cross-state analysis to examine the extent to which credit loss varies across states and whether state transfer policy has any bearing on credit loss.

One possible reason for the disconnect between statewide transfer and articulation policies and transfer student outcomes is that these policies may serve as a necessary but insufficient foundation for facilitating the transfer and application of student credits from one institution to another. For example, associate’s degrees included in many states’ transfer policy are in subjects such as liberal arts or general studies (Education Commission of the States 2016). Whereas students who earn these degrees may be guaranteed to have their credits transferred to a 4-year, they may not be assured that the credits count toward the program of study or credential they seek to attain. Students who experience this situation are required to take additional prerequisite courses depending on their chosen major. In a qualitative study that consisted of interviews with roughly 170 transfer students in Indiana, students reported that “the elective category is a kind of academic graveyard where students essentially bury all those courses that transfer but do not meet any specific requirements in the new institution” (Kadlec and Gupta 2014, p. 7). It is this concern that led an increasing number of states and institutions to develop associate’s degrees aligned with specific majors guaranteed to meet prerequisite requirements for that program at the receiving institution (Hodara et al. 2017). There is at least some rigorous evidence that the adoption of major-specific associate’s degrees may promote transfer (Baker 2016), although the effect of these policies on credit loss has not been examined.

Finally, statewide funding mechanisms for higher education may also influence transfer and credit mobility. Historically, states have funded colleges and universities primary based on student enrollments. This may create an incentive for community colleges to encourage students to take additional credits at the community college or complete their associate’s degree, even if those credits are unlikely to transfer. An increasing number of states have begun funding colleges based on student outcomes, known as performance- or outcomes-based funding (Snyder and Fox 2016; Tandberg and Hillman 2014). States such as Ohio and Tennessee have included transfer-out rates in performance funding calculations, and college administrators from those states reported that the policy increased institutional efforts to bolster transfer and articulation (Dougherty et al. 2014). These policies may reduce the incentives for community colleges to retain students with transfer aspirations, which may thereby reduce excess credit accumulation.

## Institutions

Although state policy often provides the overarching framework for transfer and articulation, institutions play a critical role in facilitating transfer and credit mobility. From the institutional perspective, transfer and articulation agreements serve two goals: to facilitate the transfer and applicability of credits, and to maintain the integrity of degree plans. While excess credit loss may negatively impact transfer students’ chances of persistence and attainment, the absence of credit loss entirely could also be a negative outcome to the extent it reflects institutions’ blanket acceptance of any and all credits for meeting degree requirements. Thus, institutions must balance their desire to recognize the prior coursework students’ have completed with the need to ensure students have in fact gained the proficiencies signified by the degree they intend to complete.

As discussed previously, the sector and control of institutions students transfer to and from is a key determinant of credit loss (US GAO 2017), likely due to the fact that the majority of statewide transfer and articulation policies focus on ensuring that 2-year credits transfer to public 4-year institutions and often exclude lateral transfers and transfer from private colleges (Ignash and Townsend 2000). This suggests the risk of credit loss may continue to grow given the increasing complexity of students’ transfer patterns (Adelman 2006; Townsend 2001).

In states with less robust transfer policy, it is up to college personnel to develop and maintain transfer and articulation policies with other institutions. Hodara et al. (2017) multi-state research identified the challenges for community colleges and their students in these institution-driven states, such as North Carolina and Texas. College personnel in these states reported having to manage thousands of articulation agreements given that they are specific to degree plans within institutions. This may result in community college personnel being insufficiently informed about what credits transfer and apply, which can in turn increase the risk that students complete courses unlikely to transfer. Students often report feeling that their community college advisors do not have the knowledge or capacity to help them effectively navigate the transfer process (Townsend and Wilson 2006), which is likely exacerbated in institution-driven states.

In addition to simply developing and maintaining articulation agreements, institutional resources likely play a critical role in facilitating student transfer. Data from NACADA’s 2011 National Survey of Academic Advising showed that the median student-to-academic advisor ratio at community colleges was 441:1, and the 25th and 75th percentile of this ratio were 184:1 and 1000:1, respectively (Carlstrom and Miller 2011; Robbins 2013). Additionally, the median ratio of students per full-time professional advisor was 1500:1 at community colleges (Carlstrom and Miller 2011). These figures reflect the tremendous range of advising resources available to community college students at different institutions, which have been found to predict students’ transfer and attainment outcomes (Bahr 2008). Research has also found that students are less likely to transfer the more they are exposed to part-time or adjunct faculty (Calcagno et al. 2008; Eagan Jr. and Jaeger 2009) and the larger the size of the institution (Calcagno et al. 2008).

Scholars have also put forth the concept of transfer receptive culture (Jain et al. 2011) as a key component of transfer student outcomes. This institutional culture consists of prioritizing and normalizing transfer and providing outreach and resources to students pre-transfer, and offering additional academic and financial supports to students post-transfer. Some reformers have recommended community colleges transform institutional culture from taking a “cafeteria style” approach to curricula to offering highly structured pathways that allow students to more easily navigate programs and complete credits with greater alignment to their educational goals, including transfer (Bailey et al. 2015). Institutional culture is also about a focus on promoting equity in transfer student outcomes, given that most state policy in transfer is “race-blind” (Chase et al. 2014) and rarely prioritizes transfer for socioeconomically disadvantaged students. Although not discussed by Jain et al. (2011), 4-year institutional culture also varies in terms of the value and respect given to community colleges, and specifically whether community college courses are perceived to be equivalent in content and rigor to courses at the 4-year. Overall, a number institutional characteristics have been found to be related to transfer student outcomes, although additional research is needed to explore whether these factors are associated with credit loss specifically.

## Students

To the author’s knowledge, no studies have yet investigated the relationship between students’ characteristics and the magnitude of credit loss they experience. However, studies have found a number of student characteristics associated with transfer outcomes, which suggests they may also have some bearing on credit loss.

An obvious possible explanation of credit loss is the courses students complete, and specifically the program of study they were pursuing prior to transfer. Completing courses in a transfer-focused curriculum is predictive of transfer success (Hagedorn et al. 2008). One may assume that students pursuing occupational programs are less likely to transfer compared to students on more academically oriented tracks, given that applied associate’s degrees are less likely to be accepted by 4-year institutions compared to academic associate’s degrees. However, research on the relationship between technical education and transfer is mixed, with some studies showing that students with vocational associate’s degrees are roughly half as likely to transfer as those with academic associate’s (Grubb 1991; Townsend and Barnes 2001) and others estimating that students with technical majors are just as likely to transfer compared to students with academic majors, controlling for a host of demographic and academic characteristics (Kienzl et al. 2012). Either way, students with greater numbers of technical credit are at greater risk of credit loss, particularly for students transferring to 4-year institutions with fewer technical program offerings (Kinnick et al. 1998).

In addition to whether courses students completed were technical, other aspects of students’ pre-transfer academic performance may influence their propensity for credit loss (Kinnick et al. 1998). Developmental or remedial courses are generally non-credit-bearing by definition and therefore unlikely to transfer. Many university programs require students to have received grades above a certain threshold in order to receive credit for the course, suggesting credit loss may be related to pre-transfer GPA. College accreditation standards generally require universities to ensure that a certain percentage of the credits applied toward a degree were earned at the institution awarding the degree, meaning students who attempt to transfer in exceedingly high numbers of credits are unlikely to retain all of their credits.

Of particular concern for those seeking greater equity in the American higher education system is whether credit loss is associated with students’ demographic backgrounds, such as race/ethnicity and SES. Research has shown that underrepresented minority (URM) students who begin at community colleges are less likely to transfer to 4-year institutions compared to white students (Bailey et al. 2005; Crisp and Nuñez 2014) and 2-year colleges with higher shares of URM students have lower transfer rates (Gandara et al. 2012; Wassmer et al. 2004). Only 13% of low-income students who begin at community colleges transfer or complete a bachelor’s degree compared to 41% of high-income students (Bailey et al. 2005), and socioeconomic disparities hold even when controlling for other student and institutional characteristics (Dougherty and Kienzl 2006; Wang 2012). Although the possible causes of these racial and SES gaps in transfer rates are numerous, particularly important for the risk of credit loss are racial and socioeconomic differences in the utilization of advising services (Orozco et al. 2010). Students form underrepresented backgrounds may be less likely to feel valued by their institution or have a caring and trusting relationship with their college advisor, which may in turn decrease the amount and accuracy of the information they receive to navigate the processes of course selection and transfer.

Overall, a variety of state, institutional, and student level factors are likely associated with credit loss. However, a primary reason the literature base contains no studies testing a holistic conceptual framework of credit loss like the one outlined above is the lack of satisfactory datasets. Institution-specific analyses are possible as institutions regularly evaluate transcripts for credit transferability and applicability, but these results may not be broadly generalizable. Studies using nationally representative datasets such as BPS (Monaghan and Attewell 2015; Simone 2014; US GAO 2017) are promising, but the BPS does not draw representative state samples, limiting the feasibility of investigating the role of state policy in credit loss. And few states require postsecondary institutions to document and report whether the credits students earned prior to transfer were accepted by the receiving institution, limiting the possibilities of investigating institutional variation in credit loss. However, this is the context for the two states included in the current study, which provides a unique opportunity to examine how credit loss relates to students and institutions for statewide cohorts. The following section discusses the higher education contexts for Hawaii and North Carolina.

## State Contexts

As discussed previously, the two states included in this study were Hawaii and North Carolina, both of which participated in the CWID initiative. Given the central role that state policy plays in transfer and articulation and credit mobility, the following sections discuss the higher education policy contexts in the two states. This information was gathered through interviews with higher education policymakers and administrators in both states and a review of statute and policy documents as part of the CWID research agenda.

## Hawaii

The University of Hawaii (UH) system was part of the first cohort of states that received a CWID grant and began implementation in 2012. The UH system consists of seven community colleges and three baccalaureate institutions and is governed by a 15-member Board of Regents. The statewide framework for transfer and articulation policy in Hawaii is embedded in the Board of Regents Executive Policy E.5209, first implemented in 1989 and revised in 1994, 1998, and most recently 2006. Among the features of this policy is the notion that transfer among the UH campuses should be as easy as possible for students, while also honoring the independence of individual campuses curriculum, degree requirements, and policies. Smooth transfer from community colleges to the universities has been and continues to be a fundamental principle of system policy.

Recent policy has also moved the UH system away from course-by-course articulation. Historically, the University Committee on Articulation was responsible for approving articulation agreements, but policy shifted to procedures that allow for waiver of the course-by-course review. As indicated in the Memorandum of Agreement: Transfer of General Education Core Requirements in May 2010, articulation now allows for either acceptance of general education in whole or components of the general education core. That is, students who complete a core general education requirement or all general education requirements within the general education framework at one UH institution are considered as satisfying those requirements at another UH institution and course-by-course review can be waived. This policy is supported by a cloud technology solution (STAR) that interacts in real time with the underlying Student Information System that is shared by all 10 UH campuses. Thus, transferring general education courses has become an automatic process facilitated by the STAR Academic Pathway solution; this technology component is an important dimension of Hawaii’s reverse credit transfer initiative, as well as its efforts to streamline transfer more broadly.

## North Carolina

Public higher education in North Carolina consists of two systems: the University of North Carolina (UNC) system and the North Carolina Community College System (NCCCS). The UNC system consists of all 16 public 4-year institutions in the state and is overseen by the UNC Board of Governors that creates and implements policies for these institutions. The NCCCS consists of 58 public 2-year institutions and is overseen by the State Board of Community Colleges. North Carolina was also part of the first cohort of CWID states and began implementing in 2013. North Carolina began by piloting RCT policies at eight of the 16 universities and 12 of the 58 community colleges, with an additional three community colleges added shortly thereafter. Although the initiative scaled up to all public institutions in the state beginning in fall 2015, only data from campuses involved in the initial pilot was provided to the CWID research team and used for this study.

Transfer between public 2-year and 4-year colleges in North Carolina is guided by the state’s Comprehensive Articulation Agreement (CAA). This policy, initially written as a response to a legislative mandate in 1995 (HB 739 and SB 1161), creates transfer routes for North Carolina’s community college students and the University of North Carolina (UNC) system, centered around the creation of a universally-recognized block of general education courses. The proposal was approved by the State Board of Community Colleges and the UNC System in February 1996 and approved by legislation shortly thereafter. According to the policy, the CAA “applies to all fifty-eight (58) North Carolina community colleges and all sixteen constituent institutions of the University of North Carolina.” Under the CAA, students who complete an associate’s degree (specified as an AA or AS) can fully transfer this block from a community college to a 4-year institution. Additionally, the CAA guarantees admission of North Carolina’s community college graduates into one of the UNC institutions, with some stipulations (such as GPA requirements, no guarantee of certain majors, and that associate’s degrees must be AA or AS). Those who transfer with an associate’s degree are also guaranteed junior status.

## Methods

### Research Questions

The purpose of this study is to address three research questions:

1. What was the magnitude of credit loss experienced by students who transferred to a public 4-year institution in Hawaii and North Carolina?
2. To what extent did the magnitude of credit loss experienced by transfers to public 4-year institutions vary according to students’ demographic and pre-transfer academic characteristics?
3. What factors most strongly predict whether transfers into public 4-year institutions lose credits and the magnitude of credit loss they experience?

### Data

The data used in this study were originally collected as part of the national research on *Credit When It’s Due* (CWID), an initiative funded by a collaborative of foundations advancing the implementation of reverse credit transfer (RCT) policies and practices. RCT is an approach for awarding associate’s degrees to students who transferred from community colleges to baccalaureate institutions without an associate’s degree and continued their enrollment at the 4-year level en route to the baccalaureate degree. In 2012, 12 states, including Hawaii, were awarded grants to begin implementing RCT policies and practices, either statewide or in a specific higher education system in the state, and an additional three states received grants in 2013, including North Carolina. The *Implementation and Outcomes of Credit When It’s Due in 15 States* provides extensive information about the initiative (Taylor et al. 2017).

The CWID research agenda consisted of two phases of student-level data collection. Before states began implementing RCT policies, our research team conducted a baseline study to estimate the numbers and percentages of students in each state who were potentially eligible to receive an associate’s degree through RCT, the university persistence and baccalaureate attainment rates of these students, and the state and institutional policy context for RCT implementation (Taylor et al. 2013). Our team requested that states, and/or higher education systems implementing RCT within states, provide data through spring 2012 on a cohort of students who transferred to 4-year institutions in fall 2008, allowing for an investigation of four-year persistence and attainment rates.

The second data collection was for the CWID outcomes study and began once states began implementing CWID (the timing of which varied by state). The purpose of this data collection was to calculate the number of transfer students that were eligible for RCT, the number of students who received associate’s degrees through RCT, and the university persistence and attainment rates of these students. The research team collected data on transfer students who were potentially eligible for RCT (i.e., students who transferred from a CWID-participating 2-year institution and completed the credit hour residency requirement prior to transfer), as well as comparison groups of transfer students. This allowed for an investigation of outcomes (persistence and attainment) associated with receiving an associate’s degree through RCT (Taylor et al. 2017).

The categories of data requested for both the baseline and outcomes study included students’ demographic characteristics, pre-transfer academic performance (e.g. pre-transfer GPA, credits earned prior to transfer, enrolled in remedial courses), post-transfer academic performance (e.g. post-transfer GPA, credits accepted at time of transfer, semester-by-semester enrollment), and postsecondary attainment (e.g. level, subject, and semester of credentials awarded), although post-transfer academic experiences are not the focus of the present study.

To calculate the magnitude of credit loss that transfer students experienced, states needed to provide two variables in either data submission: credits earned prior to transfer and credits accepted at the time of transfer. Although these variables were requested for all states, the majority of states either did not collect this data at the state level or provided invalid data (for example, where credits accepted appeared to be greater than credits earned prior to transfer for a large number of students). Indeed, of all 15 states, only Hawaii was able to provide this data during its baseline data submission, and North Carolina was able to provide this data in the outcomes study dataset. This study therefore uses Hawaii’s baseline data and North Carolina’s outcomes study data. Follow-up interviews with representatives involved with CWID in both states suggested that the data on pre-transfer credits earned and credits accepted was regularly collected and reliable.

### Sample

The sample for Hawaii includes a cohort of students who transferred to one of the three university campuses in the UH System in fall 2008. This cohort included all transfer students, regardless of whether they transferred from a public 2-year college in the UH System, a different public 4-year institution, a private college, or an out-of-state institution. Students were defined as transfer students if they had earned at least one college credit from any postsecondary institution in the past.

The sample for North Carolina includes students who were enrolled in one of the eight UNC institutions that were part of the initial CWID pilot during the fall 2014 semester and who had previously attended a public institution in North Carolina, whether 2-year or 4-year. No transfers from private colleges or out-of-state institutions were included in the sample. Although some students in the sample transferred as early as the 1980s, 96.1% of the sample transferred in 2009 or later. The dataset includes transfers from all 58 community colleges and all 16 4-year colleges in the UNC system. The descriptive statistics for both the Hawaii and North Carolina samples are included in Table 1 below.

**Table 1:** Descriptive statistics of Hawaii and North Carolina samples

	Hawaii			North Carolina		
	N	Mean	SD	N	Mean	SD
Race/ethnicity						
Amer Ind/Alas Nat	23	0.007	0.085	197	0.007	0.083
Asian	1462	0.467	0.499	969	0.034	0.181
Black	57	0.018	0.134	4257	0.149	0.356
Latino	96	0.031	0.172	1826	0.064	0.245
Multiracial				798	0.028	0.165
Nat Haw/Pac Isl				34	0.001	0.034
Non-resident alien				162	0.006	0.075
White	928	0.297	0.457	19,546	0.686	0.464
Missing	533	0.180	0.384	707	0.025	0.156
Female	1854	0.598	0.490	15,198	0.533	0.499
Pell grant						
Non-recipient	1045	0.337	0.473			
Recipient	741	0.239	0.427			
Missing	1313	0.424	0.494			
Age	3099	24.678	6.768	28,492	26.483	7.645
Pre-transfer GPA	3075	3.073	0.517	26,439	2.875	0.887
Remediation pre-transfer	1413	0.300	0.456	5983	0.210	0.409
Credentials pre-transfer						
None	2634	0.850	0.362	17,665	0.620	0.484
Certificate	124	0.040	0.189	855	0.030	0.159
AA	341	0.110	0.314	5983	0.210	0.406
AS	93	0.030	0.161	1140	0.040	0.199
AAS	31	0.010	0.100	2849	0.100	0.299
Other				570	0.020	0.127
Bachelor’s or higher				855	0.030	0.167
*n*	3099			28,492		

### Variables

The primary outcome variables of interest are the number and percentage of credits students lost at the time of transfer. These variables were derived from the two variables representing the number of credits students earned prior to transfer and the number of credits that were accepted by the receiving institution. The number of credits lost was calculated by subtracting credits accepted from credits earned prior to transfer, and the percentage of credits lost was calculated by dividing the number of credits lost by the number of credits earned prior to transfer.

The independent variables of interest fall into one of three categories: demographic characteristics, academic characteristics, and institution (both sending and receiving). Demographic variables include race/ethnicity (according to the latest US Census definitions, where Latino students are considered Latino regardless of their race, and all other racial groups are defined as non-Latino), sex, economic status (as defined by receipt of Pell grant funding during the 2008–2009 academic year), and age at the time of transfer. Academic characteristics include the number of credits students earned prior to transfer, their GPA at the time of transfer, whether they took a remedial education course prior to transfer, whether they had earned any credentials prior to transfer, and their declared major (only for Hawaii).

In regards to institutions, the Hawaii baseline dataset did not include information on the specific institution from which students transferred, but did include dichotomous variables representing whether students transferred some specific institutional types: UH 2-year, UH 4-year, in-state private, out-of-state public, out-of-state private, or unknown. The North Carolina impact dataset did include the specific public institutions in the state that students previously attended, but the dataset did not include the last institution the student attended prior to transferring, preventing an analysis of how credit loss varied by specific sending institution. The data on prior institutions attended was therefore used to categorize students based on whether they had previously attended a NC 2-year only, a NC 4-year only, or both 2-year and 4-year institutions in the state.

### Analytic Approach

The first and second research questions were addressed using descriptive statistics. The means and standard deviations (SD) of credit loss and the distribution of credit loss were calculuated for the two state samples overall as well as by sub-groups.

The third research question was addressed through a series of statistical models. First, logistic regression was used to predict whether students experienced any credit loss. The outcome was defined dichotomously, with a value of one for students with any credit loss and zero for students with no credit loss. The sample was then restricted to only students with some credit loss, and linear regression was used to examine the factors most strongly associated with the magnitude of credit loss students experienced.

As will be shown below, a large number of students did not lose any credits at the time of transfer, and the distribution of credit loss was not normal. A log-transformed version of the number of credits lost variable was therefore used in some regression models. These models had greater fit and the residuals were more normally distributed compared to models with the non-transformed credit loss variable in Hawaii. An example of the distribution of the errors from the log-transformed credit loss variable for Hawaii is included in “Appendix A”. However, Shapiro–Wilk and Shapiro–Francia tests found the distribution of the residuals from these models was significantly different from normal in some instances. Due to this issue, and the correlational nature of the study in general, the results should be interpreted as informative but not definitive.

The regression models included variables related to students’ demographic characteristics, their pre-transfer academic characteristics, the type of institutions they attended prior to transfer, and fixed-effects for the 4-year institutions to which they transferred. Because of the small number of 4-year institutions in Hawaii, an analysis of the relationship between specific institutional characteristics (e.g. funding, percent of part-time faculty, etc.) and credit loss was not feasible.

## Results

Table 2 shows the results of the calculations of credit loss for both Hawaii and North Carolina. In Hawaii, whereas students earned 55.312 credits prior to transfer, on average, students lost 2.5 credits only, on average. This translated into students losing an average of 3.9% of the credits they brought into the receiving institution. The rates of credit loss were higher in North Carolina, with students losing 4.9 credits, on average, equivalent to an average credit loss of 7.2%. Although the rates of credit loss for both states were lower than national estimates discussed in the literature review, there were at least some instances of extreme credit loss in both states. The highest number of credits lost in Hawaii was 114, and this figure was 158 in North Carolina. Both states also had students who lost all of their credits at the time of transfer.

**Table 2:** Descriptive statistics of credit loss

	Hawaii				North Carolina			
	Min	Max	Mean	SD	Min	Max	Mean	SD
Credits earned pre-transfer	1	302	55.312	34.202	1	293	59.575	26.647
Credits accepted	0	302	52.847	32.557	0	293	54.687	25.203
Credits lost	0	114	2.466	7.457	0	158	4.887	11.789
Credits lost (% of earned)	0	1	0.039	0.099	0	1	0.072	0.159

We further analyzed the distribution of credit loss in the sample by breaking the cohort into 20 groups (ventiles) based on the number and percentage of credits they lost. Each ventile contains approximately 155 students in Hawaii and 1425 students in North Carolina. Results of this analysis reveal roughly three-quarters of the sample in both Hawaii (76.1%) and North Carolina (72.6%) lost zero credits during the transfer process (Table 3). However, the top ventiles in Hawaii and North Carolina lost at least 18 and 28 credits, on average, respectively, equating to a credit loss percentage of 33.4% and 39.7% for the two states. Thus, whereas the majority of students who transferred to a public 4-year in either state were able to transfer all of their credits without any credit loss, a non-trivial percentage of students in both states lost a sizeable number of credits.

**Table 3:** Distribution of credit loss

Percentile	Hawaii		North Carolina	
	Credits lost	Credit loss %	Credits lost	Credit loss %
5	0	0	0	0
10	0	0	0	0
15	0	0	0	0
20	0	0	0	0
25	0	0	0	0
30	0	0	0	0
35	0	0	0	0
40	0	0	0	0
45	0	0	0	0
50	0	0	0	0
55	0	0	0	0
60	0	0	0	0
65	0	0	0	0
70	0	0	0	0
75	0	0	3	0.058
80	1	0.021	7	0.123
85	3	0.058	12	0.192
90	7	0.150	18	0.276
95	18	0.334	28	0.397

We next analyzed how the magnitude of credit loss varied according to student characteristics both for the full sample (Table 4) and the subset of students who experienced any credit loss (Table 5). Although there is some variation in the magnitude of credit loss across demographic groups for the full Hawaii sample, the variation appears relatively minor. The only student subgroup that lost more than 5% of their credits, compared to the 3.9% average for the entire sample, was American Indian/Alaskan Native students. Although this subgroup’s credit loss rate (6.2%) was more than twice the rate of Black students (2.6%), less than 25 students fell into this racial/ethnic category. The credit loss percentage for all other groups was between 2.5 and 5.0%. Males and females had nearly identical percentages at 3.8% and 3.9%, respectively. Pell recipients earned a greater number of credits prior to transfer than non-recipients but actually lost half as many credits (1.509 vs. 3.036) and nearly half the percentage of credits (2.7% vs. 4.6%), on average. Older students earned nearly 30 credits more than younger students prior to transfer and lost a larger number of credits, but younger students actually lost a higher percentage of credits.

**Table 4:** Credit loss by student demographic characteristics, full cohorts

	Hawaii				North Carolina			
	Credits earned	Credits acc	Credits lost	Credit loss %	Credits earned	Credits acc	Credits lost	Credit loss %
Race/ethnicity								
Amer Ind/Alas Nat	50.466	46.302	4.164	0.062	68.649	64.929	3.719	0.043
Asian	56.355	54.218	2.137	0.034	62.137	54.218	7.919	0.110
Black	61.785	60.049	1.737	0.026	60.273	53.430	6.843	0.101
Hispanic	58.802	55.392	3.410	0.036	60.983	54.325	6.658	0.094
Nat Haw/Pac Isl					59.061	49.273	9.788	0.149
White	53.672	50.566	3.107	0.049	58.994	54.932	4.062	0.061
Multiracial					56.973	52.017	4.956	0.069
Non-resident alien					69.576	59.588	9.988	0.120
Missing	54.201	52.124	2.077	0.034	62.436	56.389	6.047	0.085
Sex								
Male	54.113	51.672	2.440	0.038	58.661	53.457	5.205	0.077
Female	56.116	53.634	2.483	0.039	60.374	55.764	4.609	0.067
Pell receipt								
Non-recipient	50.502	47.466	3.036	0.046				
Recipient	54.496	52.987	1.509	0.027				
Age category								
Under 25	46.589	44.354	2.235	0.041	53.037	48.861	4.176	0.067
25 or over	77.296	74.242	3.055	0.033	74.424	67.923	6.501	0.082

**Table 5:** Credit loss by student demographic characteristics, students with any credit loss

	Hawaii				North Carolina			
	Credits earned	Credits acc	Credits lost	Credit loss %	Credits earned	Credits acc	Credits lost	Credit loss %
Race/ethnicity								
Amer Ind/Alas Nat	68.977	60.996	7.981	0.118	76.643	59.286	17.357	0.202
Asian/Pac Isl	71.650	61.377	10.272	0.162	71.751	51.893	19.858	0.276
Black	70.593	65.094	5.499	0.082	70.053	47.563	22.490	0.331
Hispanic	86.941	67.685	19.256	0.204	69.388	51.259	18.129	0.255
Nat Haw/Pac Isl					67.250	47.063	20.188	0.307
White	65.981	55.655	10.326	0.164	66.373	50.160	16.213	0.243
Multiracial					68.077	51.125	16.953	0.236
Non-resident alien					78.806	54.657	24.149	0.290
Missing	69.853	60.713	9.140	0.150	71.256	50.965	20.291	0.285
Sex								
Male	67.943	57.666	10.277	0.158	67.314	49.791	17.523	0.259
Female	70.554	60.465	10.089	0.160	68.405	50.297	18.107	0.264
Pell receipt								
Non-recipient	68.810	57.289	11.521	0.174				
Recipient	66.662	59.917	6.745	0.121				
Age category								
Under 25	58.944	48.998	9.946	0.182	63.099	47.545	15.554	0.249
25 or Over	90.238	79.620	10.619	0.116	77.868	55.314	22.553	0.286

The disparities between racial/ethnic groups were slightly larger in North Carolina than Hawaii. White students had the lowest rate of credit loss at 6.1%, apart from American Indian subgroup that constituted only 0.7% of the sample. Black and Asian students both had credit loss rates greater than 10%, and non-resident alien students lost 12.0% of their credits. Males lost slightly more credits than females (7.7% vs. 6.7%), and in contrast to Hawaii, older students in the North Carolina sample lost a higher percentage of credits compared to younger students (8.2% vs. 6.7%).

The picture appears slightly different among the subgroup of students who experienced any credit loss. In Hawaii, these students lost 10.2 credits, on average, representing roughly 16% of the credits they earned prior to transfer, and in North Carolina students lost 17.8 credits, representing 26.1% of the credits they had earned. The variation in credit loss between demographic groups is also starker in this instance for Hawaii. Black students lost the lowest number (5.5) and percentage (8.2%) of credits among all racial ethnic groups, whereas Hispanic students lost nearly four times as many credits (19.3) and had two-and-a-half times the percentage credit loss (20.4%). Both White and Asian students had close to the average amount of credit loss. In North Carolina, Black students lost the highest percentage of credits at 33.1% compared to 24.3% for White students. Interestingly, although males had higher rates of credit loss overall in North Carolina, females had a higher percentage of credits lost among the subgroup of students that lost any credits.

The next analysis investigates whether the type of credential students earned prior to transfer had any bearing on the magnitude of credit loss they experienced (Table 6). The relationship between credentials attained prior to transfer and credit loss differs starkly between the two states. In Hawaii, students who earned no credential prior to transfer lost the greatest number of credits and lost the highest percentage of credits, despite the fact that this group earned the fewest number of credits prior to transfer, on average. Students who earned certificates experienced considerably less credit loss than students who earned no credential at all, despite having earned roughly 38 more credits, on average, than students who had earned no credential. Students who earned either of the transfer-oriented associate’s degrees (AA or AS) both experienced much less credit loss than students who earned no credential, but an interesting finding is that students who earned AS degrees earned roughly 30 more credits than students who earned AA degrees, on average. It is possible that AS degrees have greater course requirements than AA degrees in the UH System or there is better articulation and alignment between AA degrees and baccalaureate degrees. Finally, although the AAS degree is generally more vocationally oriented than the AA or AS degrees, students who earned AAS degrees experienced almost no credit loss at all (0.161 credits representing 0.2% of credits earned prior to transfer). Overall, whether students earned a credential prior to transfer had a greater bearing on the extent of credit loss they experienced than the type of credential they earned in Hawaii.

**Table 6:** Credits lost by credentials earned prior to transfer

	*N*	Credits earned	Credits accepted	Credits lost	Credit loss %
Hawaii					
None	2598	50.539	47.817	2.722	0.044
Certificate	114	88.904	86.907	1.997	0.013
AA	343	75.967	75.251	0.716	0.007
AS	81	107.632	105.815	1.817	0.014
AAS	31	80.855	80.694	0.161	0.002
North Carolina					
None	17,807	49.257	46.057	3.200	0.061
Certificate	735	79.980	68.945	11.035	0.124
AA	5916	73.686	67.397	6.289	0.074
AS	1181	77.077	70.834	6.243	0.069
AAS	2831	82.374	69.457	12.917	0.140
Other	468	77.884	69.855	8.041	0.090

In contrast, in North Carolina the subgroup with the lowest rate of credit loss was students who transferred without any credential (6.1%). Even students who transferred with AA (7.4%) or AS (6.9%) degrees lost a higher percentage of credits. Additionally, in this case we find that students who earned more vocationally oriented credentials experienced greater credit loss than those who had received academic degrees. Students with certificates lost 12.4% of their credits, and students with AAS lost 14.0% of their credits, equivalent to roughly a semester of coursework (12.9 credits).

The next analysis investigates how credit loss varied according to the number of credits students earned prior to transfer (Table 7). It should be expected that students who earned more credits would experience greater credit loss in terms of the raw number of credits, simply because they had more credits to potentially lose. This is generally what we find, but both states have some anomalies. In Hawaii, students who earned 31–45 credits lost a greater number and percentage of credits compared to students who earned 46–60 or 61–75 credits. In North Carolina, students with 1–15 credits at the time of transfer lost 12.8% of their credits, approximately the same rate as students with 76–90 credits, an unexpected finding given that students in all of the intermediate credit ranges lost between 4 and 6% of the credits. These results also suggest that students who earn higher numbers of credits are indeed at greater risk of credit loss. In both states, the credit loss rate more than doubles between the 61–75 and 76–90 credit ranges. One other difference between the states is that students in the highest credit range (120+) lost the greatest number and percentage of credits in Hawaii, but these students lost a lower percentage of credits than all other groups with more than 75 credits in North Carolina.

**Table 7:** Credits lost by number of credits earned prior to transfer

	Hawaii					North Carolina				
	*N*	Credits earned	Credits acc	Credits lost	Credit loss %	*N*	Credits earned	Credits acc	Credits lost	Credit loss %
1–15	396	6.949	6.713	0.236	0.025	1063	10.538	9.329	1.209	0.128
16–30	399	24.307	23.360	0.947	0.039	3012	25.372	24.411	0.961	0.041
31–45	422	38.273	36.487	1.786	0.047	4722	37.319	35.783	1.536	0.040
46–60	514	53.776	52.202	1.574	0.030	5177	53.388	50.313	3.076	0.058
61–75	658	66.944	65.221	1.724	0.025	7890	66.902	63.394	3.508	0.051
76–90	329	81.997	77.679	4.318	0.053	3592	82.369	71.652	10.717	0.130
91–105	146	97.390	91.212	6.178	0.063	1679	96.628	82.511	14.117	0.145
106–120	77	112.561	106.807	5.755	0.050	649	112.151	94.292	17.859	0.160
120+	158	148.726	136.433	12.293	0.081	708	139.013	124.296	14.716	0.109

The previous analyses descriptively examined the magnitude of credit loss that transfer students experienced and the extent to which credit loss varied according to students’ demographic and academic backgrounds. The following analyses use logistic and linear regression methods to estimate which student characteristics are most strongly related to the the likelihood of experiencing any credit loss and the magnitude of credit loss students experienced. The outcome variable in the first (logistic) model is the dichotomous indicator of credit loss, and the outcomes in the next two (linear) models are the raw and log-transformed total credits lost variable, respectively. The reference categories excluded from the models are noted in parentheses next to the variable names. The results of the models for Hawaii are included in Table 8 and the results for North Carolina are found in Table 9.

**Table 8:** Regression models of credit loss, Hawaii

	Any Credit Loss		Credits Lost		Credits Lost (Log)	
	OR	SE	β	SE	β	SE
Race/ethnicity						
Amer Ind/Alas Nat	3.130**	1.521	0.036	3.100	− 0.321	0.295
Asian	1.059	0.124	0.849	0.911	0.111	0.087
Black	0.802	0.266	− 5.353**	2.555	− 0.640***	0.243
Hispanic/Latino	0.595	0.185	5.590**	2.643	0.183	0.251
Missing	1.057	0.156	− 0.279	1.184	0.024	0.113
Female	1.042	0.104	− 0.122	0.793	0.033	0.075
Pell recipient (yes)						
No pell	0.791*	0.106	− 3.507***	1.078	− 0.255**	0.103
Unknown	0.918	0.103	− 0.437	0.897	0.029	0.085
Age category (< 25)						
25 or over	0.931	0.117	0.135	0.991	− 0.032	0.094
Unknown	2.069	2.352	2.234	10.627	− 0.447	1.011
Pre-transfer credits (< 16)						
16–30	2.294***	0.560	2.453	2.277	0.537**	0.217
31–45	3.723***	0.877	3.902*	2.193	0.590***	0.209
46–60	3.369***	0.786	5.115**	2.160	0.656***	0.206
61–75	5.642***	1.316	7.003***	2.145	0.765***	0.204
76–90	9.451***	2.384	12.076***	2.201	1.196***	0.209
> 90	17.939***	4.694	19.248***	2.189	1.662***	0.208
Pre-transfer GPA	0.831*	0.084	0.755	0.797	0.008	0.076
Remediation pre-transfer	0.672***	0.100	− 1.398	1.347	− 0.212*	0.128
Pre-transfer attainment						
Certificate	0.534**	0.170	5.944**	2.841	0.404	0.270
AA	0.370***	0.085	− 6.040***	2.208	− 0.173	0.210
AS	0.628	0.209	− 8.030***	2.887	− 0.356	0.275
AAS	0.492	0.285	− 6.430	5.331	− 0.790	0.507
Number of sending institutions	1.044	0.052	− 0.643*	0.384	− 0.010	0.037
Sending institution (UH 2-year)						
UH 4-year	0.498***	0.100	− 3.014*	1.604	− 0.263*	0.153
In-state private	0.123***	0.028	− 1.158	1.654	− 0.382**	0.157
Out-of-state public	3.402***	0.429	1.900**	0.940	0.320***	0.089
Out-of-state private	2.137***	0.320	− 1.043	1.067	0.018	0.102
Receiving institution (UH A)						
UH B	3.126***	0.365	− 4.939***	0.884	− 0.692***	0.084
UH C	2.526***	0.456	− 8.902***	1.455	− 1.279***	0.138
Constant	0.056***	0.022	3.242	3.240	0.974***	0.308
n	3075		746		746	
Pseudo- or Adjusted R2	0.200		0.291		0.318	

*p < .10, **p < .05, ***p < .01

**Table 9:** Regression models of credit loss, North Carolina

	Any credit loss		Credits lost		Credits lost (log)	
	OR	SE	β	SE	β	SE
Race/ethnicity (White)						
Amer Ind/Alas Nat	0.743	0.184	− 1.303	1.917	− 0.211*	0.123
Asian	1.876***	0.170	1.273*	0.662	0.134***	0.042
Black	1.050	0.060	2.582***	0.418	0.153***	0.027
Latino	1.187**	0.084	0.514	0.515	0.039	0.033
Nat Haw/Pac Isl	1.068	0.116	− 0.126	0.827	− 0.001	0.053
Multiracial	1.958	0.925	0.458	3.043	− 0.065	0.195
Non-resident alien	1.777***	0.376	2.944**	1.486	0.256***	0.095
Missing	0.989	0.116	1.465*	0.882	0.115**	0.056
Female (Male)	0.704***	0.026	− 0.255	0.285	-0.003	0.018
Age 25 or over	0.641***	0.027	0.268	0.315	0.006	0.020
Pre-transfer GPA	0.909***	0.019	− 0.678***	0.168	− 0.056***	0.011
Remediation pre-transfer	14.266***	0.606	1.937***	0.295	0.247***	0.019
Pre-transfer credits (< 16)						
16–30	0.524***	0.062	1.555	1.083	0.105	0.069
31–45	0.632***	0.070	3.026***	0.997	0.297***	0.064
46–60	0.848	0.091	6.675***	0.968	0.610***	0.062
61–75	0.976	0.105	8.448***	0.958	0.669***	0.061
76–90	2.381***	0.270	17.413***	0.986	1.403***	0.063
91–105	2.233***	0.284	27.226***	1.059	1.802***	0.068
106–120	1.951***	0.312	39.626***	1.244	2.167***	0.080
120+	1.037	0.178	57.511***	1.421	2.471***	0.091
Pre-transfer attainment						
Certificate	1.025	0.114	− 1.070	0.809	0.000	0.052
AA	0.953	0.047	− 5.392***	0.379	− 0.334***	0.024
AS	1.077	0.089	− 6.156***	0.659	− 0.374***	0.042
AAS	2.082***	0.137	4.138***	0.520	0.067**	0.033
Number of sending institutions	0.395***	0.016	0.137	0.337	− 0.015	0.022
Sending Institution Type (2-Year)						
2-Year and 4-Year	0.739***	0.050	− 0.607	0.621	− 0.030	0.040
Receiving institution (NC A)						
UNC B	0.401***	0.031	− 2.105***	0.660	− 0.154***	0.042
UNC C	0.493***	0.034	− 3.839***	0.545	− 0.192***	0.035
UNC D	0.568***	0.055	0.884	0.724	0.026	0.046
UNC E	0.648***	0.048	− 4.402***	0.575	− 0.281***	0.037
UNC F	1.237***	0.075	0.550	0.443	0.069**	0.028
UNC G	0.903	0.063	− 0.888*	0.529	− 0.045	0.034
UNC H	0.448***	0.037	− 3.705***	0.702	− 0.104**	0.045
Constant	4.468***	0.678	7.429***	1.322	1.697***	0.085
n	23,220		7204		7204	
Pseudo- or Adjusted R2	0.311		0.497		0.404	

*p < .10, **p < .05, ***p < .01

In Hawaii, American Indian/Alaskan Native students were more likely to experience credit loss than White students, while Black and Latino students lost fewer credits than White students among those who experienced any credit loss. Pell recipients experienced less credit loss than non-Pell recipients, a statistically significant difference in all models. There were no significant differences in credit loss based on sex or age at the time of transfer.

In regards to pre-transfer academic characteristics, the number of credits earned prior to transfer was positively and significantly associated with the likelihood and magnitude of credit loss in all models. Pre-transfer GPA was inversely associated with the probability of credit loss but had no relationship with the magnitude of credit loss students experienced. Interestingly, students who completed remedial courses prior to transfer were less likely to lose credits and lost fewer credits than students who did not, despite the fact that remedial courses generally do not provide students with college credit. It may be the case that students are unable to “lose” these credits given that they are not awarded college credit for the courses in the first place. The results of the regression analysis generally support the descriptive finding that students who earn any credential are less likely to lose credits compared to students with no credential, with one exception. While earning a certificate pre-transfer was associated with lower odds of experiencing credit loss, among students who lost any credits earning a certificate was positively associated with the number of credits they lost.

Three findings related to the estimates of institutional characteristics in the Hawaii models are particularly noteworthy. First, students from UH 4-year institutions actually lost fewer credits compared to students transferring from UH 2-year colleges. Although it was predicted that transfers from other UH 4-year colleges would not lose substantially more credits than transfers from UH 2-year institutions given the integrated nature of UH’s transfer and articulation policy, it was not expected that transfers from 4-year institutions would have lower rates of credit loss. Second, students from out-of-state and unknown institutions had significantly higher rates of credit loss compared to their in-state transfer peers. Finally, despite the fact that the UH System appears to be integrated and coordinated in terms of its transfer policies, practices, and technological solutions, the results suggest significant variation between campuses in terms of credit loss even when controlling for student and sending institution characteristics.

In North Carolina, many of the findings from the descriptive analyses are also born out in the regression models. Asian, Black, Latino, and non-resident Alien students were either more likely to lose any credits or lost a greater number of credits compared to White students in at least one model. Females were significantly less likely than males and older students were significantly less likely than younger students to lose credits, but gender and age differences in the number of credits lost was not significant.

The findings of the relationship between academic characteristics and credit loss are different in North Carolina compared to Hawaii. In North Carolina, GPA was inversely related to credit loss in both models, whereas it was unrelated to the number of credits lost in Hawaii. Students who took remedial coursework had 14 times the odds of any credit loss than their peers in North Carolina, and remediation increased the magnitude of credit loss experienced by students who lost any credits. Although the descriptive analyses suggested that students who earned certificates lost more credits than students with no credential, these estimates were not significant in the regression model. However, the AAS degree was positively and significantly related, and the AA and AS degrees were inversely and significantly related, to credit loss in both models. These findings suggest that students pursuing occupational pathways may be at greater risk of credit loss.

In terms of the relationship between institutions and credit loss, one unexpected finding is that students were less likely to experience credit loss for every additional public postsecondary institution in the state they attended prior to transfer. Although student “swirl” is believed to be a contributor to credit loss, this may be less relevant in this instance for North Carolina given that the dataset consists only of students attending in-state public institutions. The models did not include the variable representing the type of sending institution students attended given that students who only attended public 4-years prior to transfer experienced no credit loss, but the models do include fixed-effects for receiving institution. Once again we find significant differences between campuses in their rates of credit loss when controlling for student characteristics, as many of the institutional fixed effects were significant in both the models of any credit loss and the magnitude of credit loss students experienced.

## Discussion

Enrolling in and completing college at the same institution may be part of the traditional model of higher education enrollment, but non-traditional pathways are increasingly becoming the norm (Adelman 2006; Borden 2004; McCormick 2003; Townsend 2001). Although transfer has been a central component of the postsecondary system since the origin of public junior colleges in the early twentieth century (Diener 1986) and the majority of states have a policy framework designed to facilitate transfer (Millard 2014), the frequency and types of transfer appear to be increasing (Hossler et al. 2012). This recognition is compelling states, postsecondary institutions, and higher education researchers to investigate the actual pathways students are taking through higher education to facilitate smoother transfer processes for students and address potential barriers to completion.

Despite limited research on credit loss, the most current research using national datasets has estimated that a majority of students lose at least some credits at the time of transfer, and only about half of transfer students are able to bring in all or nearly all of their credits to the receiving institution (Monaghan and Attewell 2015; Simone 2014; US GAO 2017). Indeed, some research has estimated that more than a third of transfer students lose all of their credits upon transfer (Simone 2014; US GAO 2017). Although beginning at a community college before transferring to a university to complete a bachelor’s degree may be an efficient and cost-effective approach for some students (Belfield et al. 2017), these savings are likely lost for students who lose significant portions of their credits. Given that only a limited number of studies have quantitatively examined the magnitude of credit loss that students experience, little is known about the student and institutional factors most strongly associated with credit loss and the extent to which credit loss varies across states.

The results of this study therefore contribute a number of unique insights to the credit loss literature. Perhaps the most evident is that the magnitude of credit loss in both states in the study was substantially lower than the national estimates. It is not immediately clear whether this difference is driven by methodological approach, particularly in the use of administrative data from state higher education systems in the current study compared to the use of national longitudinal data in some of the recent credit loss studies. However, this finding could also be influenced by the fact that public higher education in both Hawaii and North Carolina is regulated by a single governing board, which oversees both the 2-year and 4-year sectors, and both states also have state-level policies such as transferrable cores and transferrable associate’s degrees. Given the unique characteristics of the two states under study, generalizations should not be made about the magnitude of credit loss experienced by transfer students in other states or nationally. Conducting similar analyses in other states, particularly those with more decentralized higher education systems, would allow researchers to further explore how characteristics of state and system policy environments may relate to the transfer and applicability of credits.

Given that students from historically underrepresented groups are disproportionately more likely to begin postsecondary at a community college, this study also investigated whether any equity gaps existed in credit mobility. This did not appear to be the case in Hawaii, as there was limited variation in credit loss stemming from race/ethnicity, Pell eligibility, or gender. However, in North Carolina students of color were significantly more likely to experience credit loss compared to White students. Although the cause of these disparities is unknown, it may be the case that students from underrepresented groups may need additional support to navigate which courses to take to ensure that they transfer and apply to their chosen major (Jain et al. 2011; Orozco et al. 2010).

Another critical difference between the two states was evident in the relationship between pre-transfer credential attainment and credit loss. One possible source of credit loss is that students at 2-year institutions are increasingly pursuing technical or vocational pathways prior to transfer (Grubb 1991), and these technical courses may not transfer and apply toward a baccalaureate degree. This was the pattern found in North Carolina; students who earned certificates or applied associate’s degrees experienced even greater credit loss than their peers with no credential, whereas students who earned academic or transfer-oriented degrees lost fewer credits. However, this pattern was reversed in Hawaii, with students who had earned certificates exhibiting the lowest incidences of credit loss. Examining the policies and practices employed by the UH System to facilitate the transfer and applicability of technical credits may provide insights that can be used in other states with less alignment between technical programs at 2-year colleges and baccalaureate programs at 4-year institutions.

Although both states appear to have a high degree of coordination in their postsecondary systems, the finding that institutions differed significantly in their rates of credit loss suggests that 4-year institutions do have autonomy over transfer and articulation even in highly coordinated systems. Additionally, given the magnitude of credit loss for students from out-of-state privates in the case of Hawaii, the results suggest that UH 4-year colleges are not indiscriminately accepting credits from any and all institutions. Rather, a more likely explanation is that 4-year colleges in the UH System have robust transfer and articulation agreements with both other 4-year and 2-year colleges within the System, as well as private colleges in Hawaii. Still, institutions may choose to adopt different policies and/or practices that have a differential impact on credit loss for transfers within the system. Extending the framework of institutions’ transfer receptive culture to the study of credit transferability appears to be a potentially fruitful line of research (Jain et al. 2011).

Finally, future research should continue to investigate the intersection between state, system, and institutional policy and practice as they pertain to credit. Studies have assessed the influence of state transfer policy on transfer rates (Anderson et al. 2006) and baccalaureate attainment rates of transfer students (Roksa and Keith 2008) and found limited impact of state transfer policy on these outcomes. However, as Roksa and Keith (2008) noted, transfer policy is primarily designed to facilitate credit mobility and applicability rather than to promote transfer. Cross-state analyses correlating credit loss rates with transfer policies may help to identify which policies, if any, are effective at reducing credit loss. Additionally, interventions designed to improve the transfer, persistence, and attainment rates of college students, such as reformed advising models and technological solutions, should be evaluated in terms of their ability to reduce excess credit loss experienced by transfer students.

## Appendix A: Distribution of Residuals for Linear Regression with Log-Transformed Credit Loss Dependent Variable

Notes: The figure shows the distribution of residuals from the linear regression model of the log-transformed credit loss variable for the Hawaii sample. A normal curve is overlaid on the histogram of residuals.

Notes: The figure shows the distribution of residuals from the linear regression model of the log-transformed credit loss variable for the North Carolina sample. A normal curve is overlaid on the histogram of residuals.
